# Efficacy of a Mobile Texting App (HepTalk) in Encouraging Patient Participation in Viral Hepatitis B Care: Development and Cohort Study

**DOI:** 10.2196/15098

**Published:** 2020-04-01

**Authors:** Chul Hyun, Joseph McMenamin, Okhyun Ko, Soonsik Kim

**Affiliations:** 1 The Center for Viral Hepatitis Englewood, NJ United States; 2 W Medical Strategy Group Englewood Cliffs, NJ United States; 3 Korean Community Services Public Health and Research Center New York, NY United States

**Keywords:** chronic hepatitis B, hepatitis B virus infection, linkage to care, mobile texting app, remote consultation

## Abstract

**Background:**

Chronic hepatitis B virus (HBV) infection is a major cause of liver-related morbidity and mortality among Asian Americans in the United States. Despite the available resources, a majority of HBV-infected individuals are not able to access adequate health care owing to numerous barriers.

**Objective:**

This study aimed to assess the efficacy of a newly developed mobile texting app (*HepTalk*) in overcoming these barriers and improving patient engagement and health care access among HBV-infected and nonimmune individuals.

**Methods:**

*HepTalk* was employed for two-way communication between participants and patient navigators. A total of 82 Korean American participants who were either HBV infected or nonimmune to HBV, identified from a community hepatitis B campaign in New York, were enrolled in the study. After informed consent was obtained, both the frequency and themes of the text messages were evaluated. The effects of this communication on linkage to care at the end of the 6-month intervention period were analyzed and discussed.

**Results:**

On average, patient navigators sent and received 14 and 8 messages per participant, respectively, during the 6-month period. The themes of the messages were similar to the following 4 categories: finding providers, scheduling appointments with providers, health education, and financial issues. Of the 82 participants, 78 were linked to care within 6 months (a 95% linkage rate).

**Conclusions:**

*HepTalk* may be employed as an effective and strategic tool to facilitate communicative interaction between patients and patient navigators or health care providers, thereby improving patient engagement and health care access.

## Introduction

### Background

Patient communication with health care providers (HCPs) is crucial for positive health outcomes. Numerous barriers, however, can hinder the process of accurate and timely communication between patients and HCPs. For example, often, well-meaning HCPs provide patients medically accurate information, but unintentionally phrase it in a way that those with limited English proficiency may have trouble understanding, thus failing to adequately foster patients’ engagement with their care [1-3]. The high cost of visiting a physician may deter some patients from seeking or keeping appointments [4-7]. Outside of scheduled appointments, HCPs have only limited methods of providing patients with information and educational materials related to their health problems. Furthermore, patients may be located too far from HCPs and thus are unable to access care when the need arises [8,9]. Accordingly, there is a need for efficient communication systems or methods that can facilitate two-way communication between patients and HCPs.

Given the need for linguistically and culturally competent care in today’s diverse community, communication between patients and HCPs can be even more challenging when it involves patients and HCPs of different races and ethnicity [3,10]. Chronic hepatitis B (CHB) is a major cause of liver-related mortality and morbidity worldwide, causing numerous clinical challenges in adequate treatment and management. CHB is also a good example of health disparities whose management is complicated by a lack of adequate communication between patients and HCPs speaking different languages and from different cultures [10-12].

There is a marked ethnic and racial disparity in the prevalence of CHB in the United States. Of 2 million Americans with CHB, a majority consists of immigrants from Asia, Africa, and other parts of the world [12,13]. Specifically, 3% to 10% of Asian Americans, of whom a large majority are immigrants from China, Korea, and Southeast Asia, have chronic hepatitis B virus (HBV) infection compared with less than 0.2% of non-Hispanic white Americans. CHB management is especially critical in Asian Americans for a number of reasons. A majority of Asian Americans with CHB have been infected since their birth and have a high probability of remaining chronically infected. Among all population groups in the United States, Asian Americans are most likely to develop liver cancer [10]. Despite these risks, many people with CHB are not accessing care largely because of the absence of symptoms and the barriers that prohibit their access [12,13]. As CHB is prevalent among immigrant populations, many of whom are not proficient in the English language, serious language barriers impede communication with providers and accessing health care in the United States [10-12]. Two major issues burden CHB management in the United States: (1) a significant percentage of the population at risk is not vaccinated; and (2) a majority of chronically HBV-infected individuals, many of whom may require antiviral treatment, are not currently accessing care.

We have previously investigated the use of mobile text messaging in facilitating the connection between HCPs and individuals with CHB or at risk for it [14]. In this pilot study involving 32 participants, the frequency and contents of the messages between participants and patient navigators were evaluated. The results of the study, which showed a strong positive outcome in conduits to care after only a 3-month intervention period, suggest that mobile text messaging intervention could provide a platform for patients to engage with HCPs, thereby potentially improving their access to health care and their understanding of their own needs.

### Objective

To further examine the efficacy of mobile texting in engaging patients in collaborating in their own viral hepatitis B care, we developed a text messaging app, *HepTalk*. We evaluated the effects of its use in two groups of individuals similar to those in our previous study: people with CHB, who are not currently accessing care and thus need to see HCPs for further evaluation; and nonimmune individuals who need vaccination at a health care facility.

## Methods

### App Development

A mobile texting app called *HepTalk* was developed for a communication channel resistant to hacking that facilitates two-way communication between patients and HCPs. *HepTalk* permits encrypted texting between providers and consenting patients. Data so generated are retained throughout the period when patients are connected to providers and for 1 additional year, after which they are destroyed. Typically, the communication channel *HepTalk* is implemented as an app on an electronic device such as a smartphone or other computing device and may be used to facilitate consultation, patient navigation, and education. It may also be used for instant message or texting communication between patients and HCPs. In this manner, the secure communication channel may educate, engage, and empower patients and improve their access to HCPs.

### Hacking-Resistant Communication Channel

**Figure 1:**
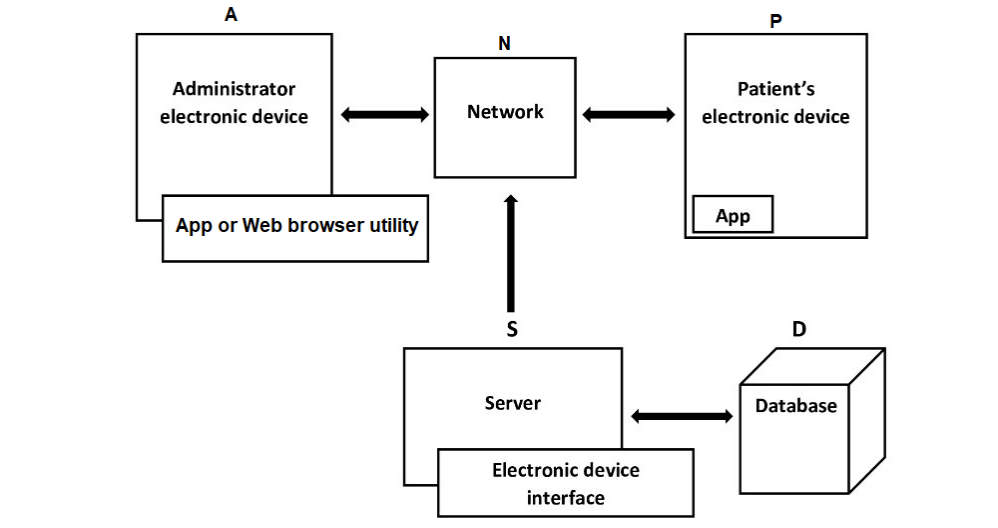
Functional diagram of a system providing a hacking-resistant communication channel through the app *HepTalk*. A: administrator electronic device; D: database; N: network; P: patient electronic device; S: server.

As shown in the functional diagram in Figure 1, the system includes the following components: a server (S) having an electronic interface, an administrator electronic device (A) with an application on a Web browser utility, and a patient electronic device (P) such as a smartphone with an app. All these electronic devices (S, A, and P) are communicatively coupled via a network (N). S is configured to generate a hacking-resistant communication channel between the app on the administrator’s electronic device and the app on the patient’s smartphone. The communication channel is set up in accordance with settings stored on a database (D), and S stores a transcript of communication on the secure communication channel on D.

### Downloading the App and Logging In

*HepTalk* is provided a link on the Apple and Android app stores. Patients may thus download the app onto their own smartphones. Once the patients download *HepTalk* app and provide log-in information to the app, the server (S) notifies the administrator (A) for approval. The administrator may approve or disapprove the patient. The approval process may include screening the patient to determine if he has the indicated medical condition. Once the patient is approved, the administrator may match the patient with an HCP (or patient navigator) and create a hacking-resistant communication channel.

### Participant Recruitment

Participants in a community-based hepatitis B awareness campaign run in Queens, New York, between January 2017 and June 2017, were Korean American adults who were found to be either nonimmune (nonimmune group) to HBV or hepatitis B surface antigen seropositive (CHB group). Korean Americans refer to those with Korean ancestry or those born in Korea but living in the United States. The campaign consisted of community-based hepatitis B screening and education organized by the Center for Viral Hepatitis (CVH) and Korean Community Services. We identified a total of 75 nonimmune participants and 28 participants with CHB not linked to HCPs.

A total of 82 (61 from the nonimmune group and 21 from the CHB group) participants agreed to participate in this study. Before the study, participants signed an informed consent form available in both Korean and English. In addition, a self-administered survey was used to determine demographic characteristics. The items in the survey included gender, date of birth, country of birth, contact information, years of residence in the United States, and preferred language. All participants preferred to communicate in Korean. Once the participants logged into the app and were linked to patient navigators, text communication began, and the messages were counted and analyzed.

### Data Collection and Analysis

All participants owned smartphones with text messaging capability. Once the participants were logged in, texting communication was initiated by the patient navigators. Members of the nonimmune group were sent an initial message advising them to receive hepatitis B vaccination at a local health care facility and asking at the same time if the participant needed help in locating such a facility. Members of the CHB group were also sent initial messages advising them to see physicians for further evaluation of HBV infection. The participants in the latter group were also given a list of local health care facilities and doctors’ offices where they could potentially receive further care.

The frequency of text messaging to and from the patient navigators was recorded. The contents of the messages were then analyzed by classifying them under 1 of the 4 following thematic categories: medical access (finding physicians or health facilities), reminders and schedules, financial costs and insurance, and health information and education. Medical access referred to finding physicians or health facilities the participants felt comfortable visiting for evaluation or vaccination. Reminders and schedules include notifications to alert the participants to make and keep appointments. Financial costs and insurance referred to medical expense and having or lacking a health insurance plan. Finally, health information and education included all messages providing health information on CHB and prevention of HBV infection by vaccination. These included updated guidelines and recommendations on CHB available from community centers, and their current activities, and printed and Web resources (eg, US Centers for Disease Control and Prevention).

### Linkage to Care

Outcome in linkage to care (LTC) was evaluated at the end of 6 months. In the nonimmune group, the participants who received at least two hepatitis B vaccinations were considered *linked*. In the CHB group, the participants who saw a physician at least once for further evaluation of their CHB status were also considered linked to care. All the CHB participants who were linked to physicians had a hepatitis B DNA test, and their attendance at scheduled office visits was confirmed by the patient navigator.

### Patient Navigator

The patient navigator program was used to provide participants with specific information on the prevention and management of CHB and to link them to HCPs who have expertise in hepatitis B care within their community. Patient navigators are health care professionals who can assist the participants in finding their way through the health care system and who work to overcome obstacles by identifying and providing resources for Korean Americans with socioeconomic and communication barriers.

This study involved 2 patient navigators. One held a Bachelor of Science degree in public health, and the other held a Master of Public Health degree. They were employees of community service organizations and were familiar with clinicians and other health care resources within the community. The participants in the nonimmune group were provided with a list of health care facilities where they could be vaccinated. All participants in the CHB group were given a list of community HCPs with expertise in hepatitis B. Throughout all the participant office visits and appointments, patient navigators used *HepTalk* to guide the participants through the process of evaluation. Communication between subjects and patient navigators relied on text messaging (ie, *HepTalk*), and the patient navigators kept detailed records of all communication with the subjects.

### Ethics Approval and Consent to Participate

All procedures performed in studies involving human participants were in accordance with the ethical standards of the institutional and/or national research committee. The Investigative Committee on Clinical Research, institutional review board of Holy Name Medical Center, Teaneck, New Jersey, approved this study.

### Availability of Data and Materials

The datasets used and analyzed during this study are available from the corresponding author on reasonable request.

## Results

### Demographic Characteristics

Between July and December 2017, we registered a total of 82 individuals and followed them up with *HepTalk* texts. Table 1 shows the demographic characteristics of both groups. The nonimmune group consisted of 61 individuals requiring HB vaccination. Of these 61 participants, 28 were men and 33 women; the average age was 47 years. The CHB group consisted of 21 individuals who had been found to be hepatitis B surface antigen seropositive. There were 10 men and 11 women; the average age was 54 years. All the participants were Korea-born immigrants who preferred to communicate in Korean. Once registered on *HepTalk*, they were assigned to patient navigators, with whom all the participants subsequently communicated through *HepTalk*. The nonimmune group was advised to go to recommended medical facilities to receive vaccinations, whereas the CHB group was advised to see providers for further assessment and management of their CHB. All the communication between the participants and the patient navigator took place through *HepTalk* during the 6-month period between July 2017 and December 2017.

**Table 1:** Demographic characteristics and linkage to care rate after a 6-month intervention period.

Groups			Vaccine group (n=61)	Chronic hepatitis B group (n=21)
**Sex, n (%)**				
	Male		28 (46)	10 (48)
	Female		33 (54)	11 (52)
**Age (years)**				
	Mean		47	54
	**Range, n (%)**			
		21-29	8 (13)	0 (0)
		30-39	6 (10)	1 (5)
		40-49	18 (30)	4 (19)
		50-50	19 (31)	11 (52)
		60-69	8 (13)	5 (24)
		70-79	2 (3)	0 (0)

### Communication Between the Participants and Patient Navigators

Tables 2 and 3 demonstrate the number and frequency of messages sent and received by patient navigators. All participants received at least one message sent by the assigned patient navigator at the beginning of the 6-month intervention period. The patient navigators sent a total of 852 messages to members of the nonimmune group. All 61 nonimmune participants responded with a total of 463 messages, averaging 7.6 messages per respondent (Table 2). Patient navigators sent 299 messages to the CHB group, consisting of 21 participants. All, except 5 participants, responded with a total of 157 messages, averaging 9.8 messages per texting respondent (Table 3).

**Table 2:** Number of messages sent and received for group 1.

Number of messages	Number of participants receiving messages	Number of participants sending messages
1-5	15	38
6-10	19	9
11-15	9	2
16-20	6	4
21+	12	8

**Table 3:** Number of messages sent and received for group 2.

Number of messages	Number of participants receiving messages	Number of participants sending messages
0	0	5
1-5	7	7
6-10	3	3
11-15	2	1
16-20	3	4
21+	6	1

### Frequency and Common Themes of the Communication

The contents of the messages received by both groups were categorized into 1 of the following 4 themes: finding medical access, reminders and scheduling, education and health information, and financial and insurance.

The greatest proportion of the messages in both groups was related to medical access, followed by the other 3 categories, whose proportions were similar in frequency. The proportion of the messages pertaining to HCPs (medical access) and scheduling was greater than half of all the messages received (282/461, 61.2% and 85/147, 57.8% in the nonimmune and CHB groups, respectively).

However, the frequency of communication was markedly different between the nonimmune and CHB groups. Although three-fourths of the total communication in the nonimmune group was carried out within 3 months of intervention, the same proportion of the total communication in the CHB group was carried out within the first month. This difference may be related to the fact that to be considered *linked* in the nonimmune group, a participant had to have 2 vaccinations 1 month apart, whereas to be considered *linked* in the CHB group, a participant required only 1 visit to an HCP. The linkage in the CHB group was rapid, with a mean of 14 days. The most common themes of the messages involved medical access and reminders in both groups.

### High Level of Achievement in Linkage to Care

At the end of the 6-month intervention period, 78 of 82 individuals were linked to care, demonstrating an overall linkage rate of 95%. Specifically, 58 of 61 individuals in the nonimmune group received their first 2 vaccinations within an average period of 65 days. In the CHB group, 20 of 21 participants made 2 or 3 visits to their providers’ offices for the evaluation of their CHB within the 6-month period (Table 4). Those participants who accessed care in the CHB group made their first visit to HCPs within an average period of 14 days of *HepTalk* intervention.

**Table 4:** History of hepatitis B virus infection in the hepatitis B surface antigen-seropositive participants linked to care.

Years since hepatitis B virus was diagnosed	Number of participants linked to care (n=20), n (%)
<5	2 (10)
5-10	3 (15)
11-20	2 (10)
>20	13 (65)

### History of Hepatitis B Virus Infection in Chronic Hepatitis B Group

We evaluated how many of the 20 linked CHB participants had known their infection status before the current screening and *HepTalk* intervention. As shown in Table 4, 18 of the 20 participants had been aware of their infection for more than 5 years before the texting intervention began. Only 5 of these patients had seen physicians at least once in the past, but they did not return for continued care.

It is noteworthy that the remaining 15 of these linked patients had known their HBV status for over a decade and yet had not seen an HCP for further evaluation before their current linkage.

## Discussion

### Principal Findings

Secure communication via texting between patients and HCPs may be used for consultation, patient engagement and education, and direct instant messaging [15-17]. Much evidence supports the use of texting between patients and HCPs and positive health outcomes. Text messaging can provide laboratory results, reminders of appointments, medication administration and flu vaccination, and other services [18-20]. Specifically, text messaging has been shown to improve adherence to medication and attendance at medical appointments among HIV and other chronic disease patients [21,22]. Text4Health projects have also helped to engage the underserved population and improved their health [23]. SmokeFreeText, for instance, more than doubled the smoking cessation rate among teens by texting smoking cessation messages to them [24]. Furthermore, the Text4Baby Campaign has helped expectant mothers to receive crucial prenatal care resources, thereby fostering the safe delivery of their babies [25].

As texting has become a progressively effective tool in patient engagement, the number of health apps has dramatically increased during the past decade. As of 2013, there were more than 1700 diabetes mellitus apps in all the app stores combined (Apple app store, Google Play store, and Windows) [26]. These mobile health apps in smartphones can collect and deliver health care data and monitor patients’ vital signs in real time [27,28].

The results of this study demonstrate that *HepTalk* can be employed to boost patient engagement and improve outcomes in hepatitis B care. This study further supports the finding of our previous study, which suggested that a form of mobile texting combined with the patient navigator program facilitated communication between the patients and HCPs and enhanced LTC [14]. *HepTalk* provided an effective communication mechanism through which patient navigators were able to guide participants to appropriate health care resources. The benefits of *HepTalk* are enormous, including communication speed, accessibility, and reduced patient expense. *HepTalk* is not limited by geographic boundaries and is able to help people lacking transportation. Last but not least, as all the communication was in the patients’ native language, *HepTalk* could also overcome linguistic and cultural barriers.

### Rapid and Effective Patient Engagement on Patients’ Terms

Our results also demonstrated a high level of texting communication between the participants and patient navigators. First, patient navigators sent a total of 1151 messages to all 82 participants, averaging 14.0 messages per participant. Second, patient navigators received a total of 620 messages from 77 participants, averaging 8.1 messages per participant (Tables 2 and 3). All but 5 participants in the CHB group replied to patient navigators through *HepTalk*, demonstrating a 94% response rate from the participants. The 5 participants who did not reply to the patient navigators were sent 2 repeat messages advising them to see a provider, providing a list of community HCPs. Of these 5 participants, 4 were finally linked to providers in the community. Thus, texting communication took place on the participants’ terms and allowed them the flexibility to interact with the patient navigators in the manner they felt most comfortable with. Most of the messages were read and replied to within a few hours, further demonstrating the likelihood and speed of interaction.

The mean number of messages received and sent by each participant in the CHB group during the 6-month period was 14 and 10, respectively. Very few messages were sent or received once the LTC was confirmed. As the mean length of time needed to establish LTCs was only 14 days, linkage in the CHB group was rapid, suggesting patients’ motivation to engage with care. The observed high rate of LTC is remarkable, especially given that the majority of the patients in the CHB group had been diagnosed years before this campaign and had not previously accessed HCPs (Table 4).

The efficacy of *HepTalk* in participant engagement observed in this study is attributable to successful communicative interaction between the patient navigators and participants. The themes noted in the texts were diverse and covered important topics that typically would be covered between patients and HCPs in their offices. Although finding HCPs was a slightly dominant theme, the frequency of the other 3 themes, related to making and keeping appointments with HCPs, health education, and financial factors, was equal; the number of text messages during the 6-month period was the same in all, that is, 3 messages.

### Positive Health Behavior Change

It is noteworthy that the majority of the CHB patients in this study had been aware of their infections for many years before this study. Of 20 CHB patients linked to care, 18 had known their infection status for 5 years or longer, yet they had not seen HCPs for continuing care (Table 4). These results are congruent with the previous findings that only a minority of HBV-infected people could access care. According to these previous investigations, only about 40% of subjects with CHB screened in a community setting were successfully linked to care [10-13].

How did the *HepTalk* app, then, help the HBV infected participants to change their minds and access care they had failed to do so in the past? *HepTalk* combined with the patient navigator program in this study helped to overcome barriers caused by language and culture, as all texting took place in the Korean language. Furthermore, as the texting cost nothing, patients faced no financial burden. Finally, patients did not have to spend much time in finding HCPs and health care facilities and arranging medical appointments, thereby diminishing the burden of finding providers. Once patients were able to understand through effective communication (eg, same language and culture) the need for vaccination and further evaluation, they were motivated to access care. Thus, *HepTalk* helped to drive positive behavioral change in the HBV-infected participants who might otherwise have failed to address their problems in the past.

It has been well established that health behaviors can have an impact on individuals’ physical and mental health and quality of life [29,30]. Behaviors that relate to accessing care and behaviors that involve care seeking and adherence to treatment, for instance, can be crucial in determining the effective management of CHB and numerous other chronic diseases. By overcoming various barriers, *HepTalk* provided an environment designed to empower and direct the individuals with CHB to change their behaviors.

### Beneficial Features of HepTalk

The benefits of *HepTalk* are substantial, including accessibility, flexibility, ease and speed of communication, and reduced patient expense. *HepTalk* is designed specifically as a hepatitis B app to facilitate hepatitis B care. Patients can easily identify the app on the smartphone screen, allowing quick access. *HepTalk* does not rely on cellphone data plans; it can be used with Wi-Fi alone. This *Wi-Fi–compatible* feature is not only convenient as Wi-Fi is readily available in cities but also can help to reduce cost, as smartphone plans charge for texting. *HepTalk* is thus more affordable than SMS. Unlike most other apps, *HepTalk* allows communication between a patient navigator (or HCP) and only 1 patient at a time. No second party can be invited, protecting the privacy of the communication. Administration, however, can reach out to any number of patients at once to make announcements. *HepTalk* can also provide patients with unlimited access to educational material and information outside of their scheduled appointments. In addition, *HepTalk* has many features that regular SMS or texting apps lack, including an ability to file patients’ demographics and categorize the patients (eg, nonimmune and CHB groups). These capabilities allow easy and convenient access and communication between the participants and the patient navigators. Furthermore, although not available worldwide, *HepTalk* is not limited by geographic boundaries and can serve people lacking transportation. Last but not least, *HepTalk* can also overcome many issues related to cultural competence. Patients with a specified culture and language can connect with patient navigators or HCPs who share the same culture and language, thus allowing optimal communication between the parties.

One difficult aspect of *HepTalk* use from patients’ perspectives, however, is the downloading and log-in process. Unlike most other apps, *HepTalk* log-in requires approval by administration. Approval requires a process of screening the patient to determine if he or she has an appropriate medical condition. Although a few patients had difficulty with log-in, this screening process may be considered more of an advantage than a disadvantage because it allowed distinguishing individuals who need the *HepTalk* service from those who do not.

### Limitations of This Study

We have to consider important shortcomings of this study. First, the sample size (n=82) may not be large enough to reflect results to be expected among Korean Americans, in general, in the United States, whose proportion is much less than that of other ethnic groups. Although it is well known that age, education, and socioeconomic status influence patients’ health care access behavior and their uptake and adaptation of mobile technology, we have not assessed these factors among the participants enrolled in the study. Second, this study did not directly compare the effects of *HepTalk* texting on patients who refrained from *HepTalk* texting. Although we have not conducted a study where we employed a control group denied access to *HepTalk* texting, this study and previous studies have demonstrated a significant lack of LTC in people with CHB in the absence of any mobile texting interventions [10-13]. Third, our study did not compare *HepTalk* with regular SMS texting, thus limiting specific comments on direct comparison between the two. However, it should be noted that there are unique features that *HepTalk* offers, and SMS or other apps do not, which may make *HepTalk* more desirable for patients’ use (see the Discussion above). Fourth, we considered CHB participants who saw HCPs at least once during the 6-month period for evaluation with DNA testing to be linked to care. However, it may be premature to consider these participants linked because some of these *linked* participants may not return to HCPs for follow-up in the future. Thus, although the texting intervention was significantly longer in this study compared with the intervention period employed in our previous study [14], studies with an intervention period greater than 6 months may be preferable to assess the sustainability of the efficacy of the *HepTalk* communication. This is particularly important because we have noted that a small but significant portion of CHB patients with long histories of infection saw HCPs once but did not sustain their LTC. Finally, it should be noted that *HepTalk* alone would not have led to the observed successful LTC. The exact role and contribution of the patient navigator program to the overall success of *HepTalk*-mediated LTC need to be better defined. For instance, the benefit arising from the use of *HepTalk* alone cannot be distinguished from the benefit arising from the linguistic and cultural competence of patient navigator program implementation.

In conclusion, the results of this study suggest that *HepTalk* can serve as an effective communication tool that may empower patients to access health care. The patient navigator program and mobilization of local HCPs who have expertise in CHB were also crucial to successful linkage of the patients from the testing site to providers. Mobile texting combined with community-based patient navigation programs such as those employed in this study may be implemented in other minority ethnic populations to enhance hepatitis B care or, perhaps, the care of patients with other chronic illnesses. Future studies with *HepTalk* or other instant messenger apps evaluating a larger population at different economic and sociocultural levels for longer periods would be needed to better assess the efficacy and sustainability of text messaging intervention in the enhancement of LTC in hepatitis B care.

## Abbreviations

CHB: chronic hepatitis B
CVH: Center for Viral Hepatitis
HBV: hepatitis B virus
HCP: health care provider
LTC: linkage to care
